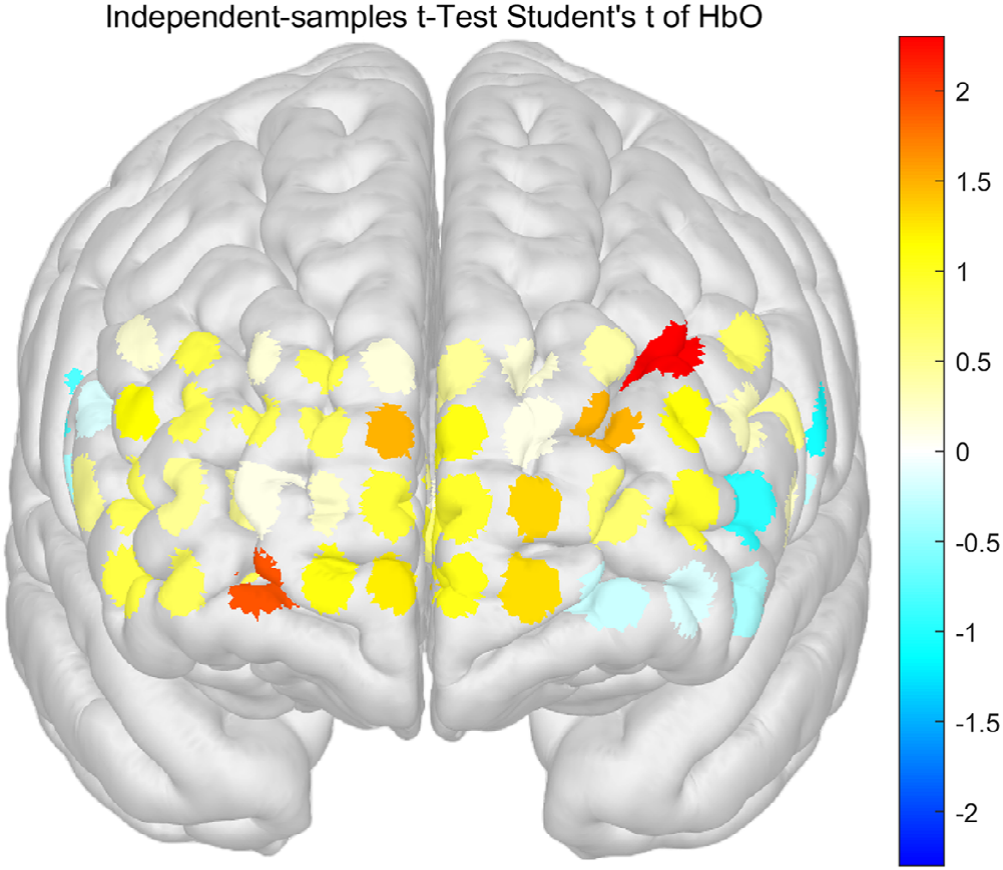# Development of the Problematic Social Network Sites Use Scale (PSNSU) and Its Psychosocial Impact on Generation Z

**DOI:** 10.1002/alz70858_098171

**Published:** 2025-12-24

**Authors:** Seungju Lim, Ji‐Hyuk Park

**Affiliations:** ^1^ Yonsei University, Wonju, Gangwon‐do, Korea, Republic of (South)

## Abstract

**Background:**

The rapid adoption of social network sites (SNSs) among Generation Z has raised concerns about their neurological, cognitive, and psychosocial well‐being. Despite growing awareness, a standardized assessment tool to measure problematic SNS use (PSNSU) is limited. This study aimed to (1) develop and validate a robust PSNSU scale with a precise cutoff score for distinguishing problematic users, and (2) examine differences in neural activity, executive function, and lifestyle between problematic and non‐problematic SNS users.

**Methods:**

A total of 254 SNS users aged 18–39 participated. The study was conducted in two phases. In Phase 1, a 42‐item Likert‐scale PSNSU questionnaire underwent exploratory and confirmatory factor analyses. Receiver Operating Characteristic (ROC) analysis determined the optimal cutoff score. In Phase 2, participants were classified into problematic (*n* = 56) and non‐problematic (*n* = 48) groups. Functional Near‐Infrared Spectroscopy (fNIRS) was used to measure dorsolateral prefrontal cortex (DLPFC) activation during Stroop tasks. Additional assessments measured lifestyle balance (YLP‐ABCD), quality of life (WHOQOL‐BREF), and executive function (Frontal Assessment Battery).

**Results:**

The PSNSU scale demonstrated excellent internal consistency (Cronbach's α = 0.947) and a strong four‐factor structure (accounting for 69.48% variance). The optimal cutoff score of 63 (AUC = 0.86) showed high sensitivity (0.844) and specificity (0.746). Problematic SNS users exhibited lower DLPFC activation, indicating impaired cognitive control. They also scored significantly lower in lifestyle balance, executive function, and quality of life assessments (*p* < 0.01), suggesting pronounced psychosocial vulnerabilities.

**Conclusion:**

The study highlights the association between problematic SNS use and deficits in neural and cognitive function, emphasizing the need for targeted interventions. In particular, the declines in lifestyle balance, executive function, and quality of life suggest that problematic SNS use impacts not only cognitive control but also overall daily functioning and psychosocial well‐being. These findings underscore the importance of comprehensive intervention strategies that address both cognitive control improvements and the lifestyle and emotional challenges related to excessive SNS use.